# Absence of a spin-signature from a single Ho adatom as probed by spin-sensitive tunneling

**DOI:** 10.1038/ncomms10454

**Published:** 2016-02-03

**Authors:** M. Steinbrecher, A. Sonntag, M. dos Santos Dias, M. Bouhassoune, S. Lounis, J. Wiebe, R. Wiesendanger, A. A. Khajetoorians

**Affiliations:** 1 Department of Physics, Hamburg University, 20355 Hamburg, Germany; 2 Peter Grünberg Institut and Institute for Advanced Simulation, Forschungszentrum Jülich & JARA, 52425 Jülich, Germany; 3 Institute for Molecules and Materials (IMM), Radboud University, 6525 AJ Nijmegen, The Netherlands

## Abstract

Whether rare-earth materials can be used as single-atom magnetic memory is an ongoing debate in recent literature. Here we show, by inelastic and spin-resolved scanning tunnelling-based methods, that we observe a strong magnetic signal and excitation from Fe atoms adsorbed on Pt(111), but see no signatures of magnetic excitation or spin-based telegraph noise for Ho atoms. Moreover, we observe that the indirect exchange field produced by a single Ho atom is negligible, as sensed by nearby Fe atoms. We demonstrate, using *ab initio* methods, that this stems from a comparatively weak coupling of the Ho 4*f* electrons with both tunnelling electrons and substrate-derived itinerant electrons, making both magnetic coupling and detection very difficult when compared to 3*d* elements. We discuss these results in the context of ongoing disputes and clarify important controversies.

## Abstract (web summary)

Magnetic stability of holmium atoms on a platinum(111) surface has recently been reported, raising prospects for atomic-scale spintronics, however contradictory results have since emerged. Here, Steinbrecher *et al.* find evidence for an invisibility of the holmium spin to scanning tunnelling spectroscopy techniques which challenges recent results.

## Graphical abstract

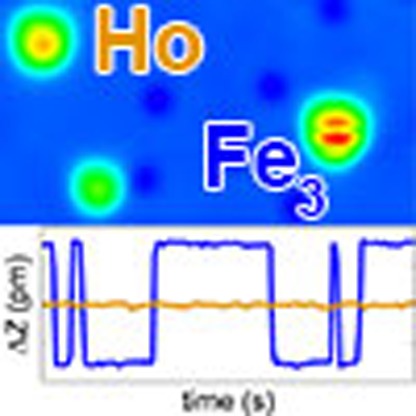

Using the magnetic moment of a single atom adsorbed on non-magnetic surfaces to store and process information is one of the major goals in the field of nano-spintronics^1^. A key ingredient towards realizing single-atom magnets is the large magnetic anisotropy energy^2^, which defines an energy barrier between various orientations of the magnetic moment and has been found to be extraordinarily large for single 3*d* atoms, like Co, adsorbed directly on metallic surfaces^3^. However, strong hybridization of the 3*d* states makes the magnetic moment susceptible to substrate-driven interactions, like spin-flip scattering by conduction electrons^4^, diminishing the stability of the magnetic moment against fluctuations. One route towards increasing the magnetic stability of single-atom magnets is to use strong exchange coupling between a small number of magnetic atoms, thereby increasing the effective energy needed to reverse the magnetization of the atomic ensemble. Towards this end, it was demonstrated that a small number of strongly coupled Fe atoms in an array^5^ or a chain^6^,^7^, either ferro- or antiferromagnetically coupled, can be controllably stabilized into a given state from seconds to hours.

It was also proposed that, by combining a particular choice of symmetry, magnetic moment and strong uniaxial anisotropy, the spin can be protected from quantum tunnelling of the magnetization and substrate-driven relaxation^8^,^9^,^10^. Experimentally, based on this concept, Miyamachi *et al.*^8^ claim to stabilize a single Ho atom on Pt(111). This realization is motivated by a key property of bulk 4*f* magnetism; the spin resides in the 4*f* orbitals, which are strongly localized to the nucleus and only couple to the itinerant electrons via intra-atomic exchange paths through the 5*d*- and 6*s*-orbitals^11^ and therefore the 4*f* orbitals only negligibly contribute to the tunnelling current^12^. This property of 4*f* elements hypothetically allows for a weaker degree of hybridization as compared with the 3*d* counterparts, when adsorbed onto metallic surfaces. However, there has been recent controversy about this claim, stemming from disagreement about the magnetic properties of Ho/Pt(111). X-ray magnetic circular dichroism (XMCD) measurements^13^ reveal no evidence of magnetic stability and a different ground state configuration for Ho/Pt(111) compared with ref. 8, which violates the theoretical criterion proposed by Miyamachi *et al.*^8^.

To clarify this controversy and understand if tunnelling-based spectroscopy can reveal the magnetism of 4*f* elements, we revisit Ho atoms on Pt(111) with spin-resolved tunnelling spectroscopy at *T*=0.3 K. In stark contrast to what is reported by Miyamachi *et al.*^8^, we see no evidence of spin-excitations or spin-polarized telegraph noise on these atoms. The findings were corroborated by concurrently performed experiments with Fe atoms on the same surface, where the magnetic properties of this system are well-known^14^ and confirm our spin-sensitivity. Furthermore, by monitoring the changes in spin-excitations of a single Fe atom, we find that the indirect exchange fields produced by a nearby Ho atom cannot be detected. These findings, as illustrated by *ab initio* methods, stem from a weak interaction of the 4*f* orbitals with the surrounding electronic environment, which thereby makes the Ho magnetic moment difficult to access by both tunnelling methods and indirect exchange pathways.

## Results

### Inelastic tunnelling spectroscopy of single Ho and Fe atoms

**Figure 1:**
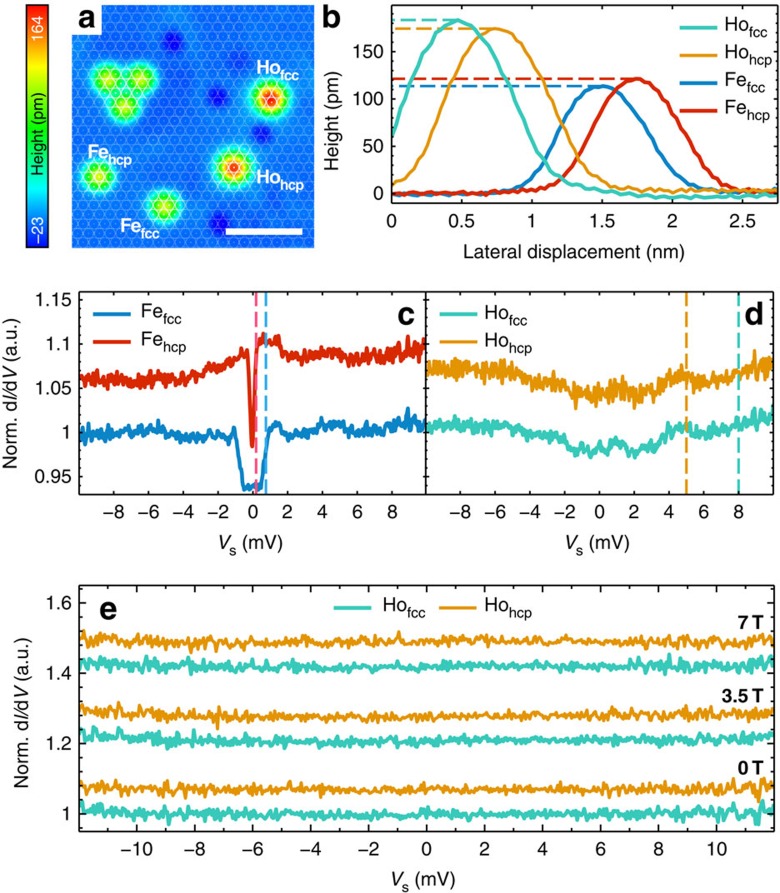
Topography and inelastic spectroscopy of a single Fe and Ho atom. (**a**) Constant-current image of an artificial arrangement of Fe and Ho atoms on fcc and hcp lattice sites (*V*_s_=10 mV, *I*_stab_=0.5 nA). The overlaying white lattice has the lattice constant and orientation of the Pt(111) substrate but is centred on the Fe_hcp_ species and reveals the different adsorption sites of the atoms. The white scale bar has a length of 2 nm and the colour scale on the left side indicates the measured apparent height in a range of −23 to 164 pm. (**b**) Line profiles of the atoms in **a** taken from a topography recorded at *V*_s_=−50 mV and *I*_stab_=100 pA. The dashed lines indicate the dedicated maximum heights. (**c**,**d**) 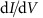 spectra measured on the isolated Fe and Ho atoms of **a** using the same microtip. The dashed lines indicate previously reported spin-excitation energies^5^,^8^ (*I*_stab_=5 nA, *V*_stab_=10 mV, *V*_mod_=40 μV, *f*_mod_=4.142 kHz, 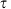
=10 ms and *V*_sens_=10 mV). (**e**) Magnetic field-dependent 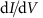 spectra measured on an isolated Ho atom at the indicated *B*_*z*_ values and settings as in **d** except for *I*_stab_=6 nA, *V*_stab_=12 mV. Spectra in **d**,**e** have been normalized by subtraction of a substrate spectrum taken with the same microtip and are artificially offset for clarity.

In constant-current scanning tunnelling microscopy (STM) images, Ho and Fe atoms can easily be distinguished by their different apparent heights, which are ≈180 pm for Ho and ≈120 pm for Fe atoms, respectively (Fig. 1a,b). We observe that the apparent height of the Ho atom depends strongly on the bias voltage *V*_s_ applied to the sample with a maximum of ≈205 pm at *V*_s_=1.5 V (see Supplementary Note 1 and Supplementary Figure 2), in excellent agreement with the results of Donati *et al.*^13^. These values significantly deviate from what was published by Miyamachi *et al.*^8^, where apparent heights above 220 pm (*V*_s_=1 V) have been reported. We find small differences in the apparent height for fcc and hcp atoms. Interestingly, the Fe_hcp_ atoms appear higher than Fe_fcc_, whereas Ho atoms show the opposite behaviour (Fig. 1b). With a given tip, inelastic scanning tunnelling spectroscopy (ISTS) on the Fe atoms reveals the previously reported^14^ adsorption-site-dependent spectroscopic signature of a spin-excitation with a different energy for Fe_fcc_ and Fe_hcp_ sites. They are located at 0.75 meV and 0.19 meV (dashed lines in Fig. 1c), with step heights of 8% and 12% of the signal at zero bias, respectively. Knowing the adsorption site, tip-induced atom manipulation has been used to assemble artificial arrangements of Ho_fcc_, Ho_hcp_, Fe_fcc_ and Fe_hcp_ on clean areas of the Pt(111) substrate, which we used for subsequent investigations of their magnetic properties (Fig. 1). Typical manipulation parameters are *V*_s_=2 mV at a stabilization current of *I*_stab_=50 nA for Fe, while Ho already moves at 30 nA.

We first performed magnetic field (*B*)-dependent ISTS and spin-resolved measurements on isolated Ho atoms which were far from other atoms. Using the same microtip as for ISTS on Fe (Fig. 1c) the differential tunnelling conductance (d*I*/d*V*) signal on Ho_fcc_ and Ho_hcp_ is featureless and shows no reproducible inelastic signal distinguishable from the substrate spectrum (Fig. 1d). In particular, there is no indication for a spin-excitation at the previously reported energies of 5 meV and 8 meV for Ho_fcc_ and Ho_hcp_, respectively^8^ (see dashed lines in Fig. 1d). Moreover, the spectra in an energy window of ±12 meV do not change in a magnetic field up to 7 T (Fig. 1e). This rules out that spin-excitations emerge in a magnetic field at low energy due to negligible magnetic anisotropy^15^. We performed the same experiment with several tens of different microtips and atoms with stabilization currents up to 50 nA and modulation voltages reaching 3 mV using a second lock-in amplifier to measure the second derivative signal in parallel to (d*I*/d*V*) (see Supplementary Note 2), to directly compare with the method used by Miyamachi *et al.*^8^. Several spectra with different parameters have been taken and are shown exemplarily in Supplementary Figures 3–5 in Supplementary Note 2. The noise level in these measurements was ±0.5 μA V^−2^ (see Supplementary Note 2). Nevertheless, we did not see any inelastic signatures unique to the Ho atom, regardless of the various parameters. It is important to note that tip-related features which appear as variations in the substrate spectra could not be fully removed from the spectra measured on the atoms by subtracting a substrate spectrum measured with the same tip. For all microtips we used, the minimum signal variation due to this effect was ±2 μA V^−2^ (see Supplementary Note 2). We remark, however, that the intensity of the inelastic signal was not specified by Miyamachi *et al.*^8^.

### Spin-resolved measurements of Ho and Fe_3_

**Figure 2:**
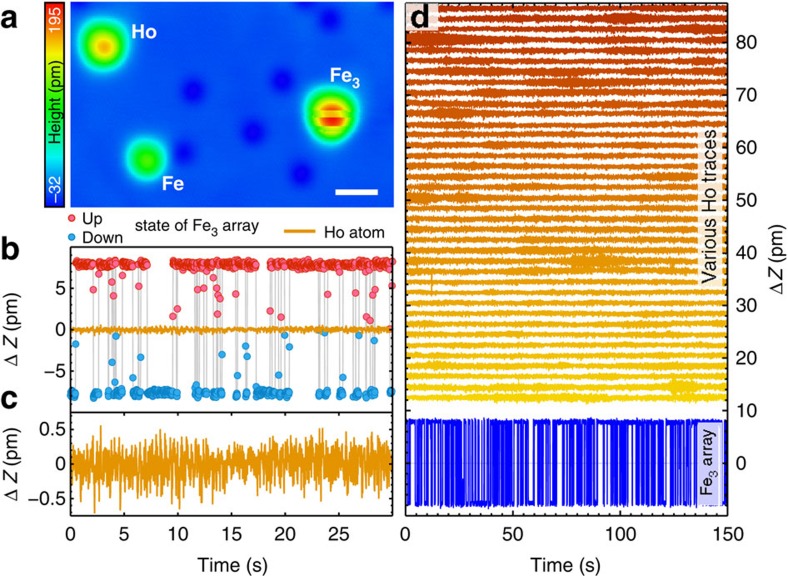
Spin-polarized measurements on single Ho atoms and an Fe_3_-array. (**a**) Constant-current image of an Fe_3_-array, an Fe and a Ho atom (*V*_s_=−5 mV and *I*_stab_=0.5 nA) recorded with a spin-polarized tip. The height of the Fe_3_-array (≈200 pm) changes randomly while recording the image due to switching of its magnetization. The white scale bar has a width of 1 nm and the colour scale on the left side indicates the measured apparent height in a range of −32 to 195 pm. (**b**) Time trace of the magnetic telegraph noise of the Fe_3_-array (colours red and blue represent the two magnetization states up and down) and of a Ho atom recorded with the same magnetic tip (orange line) (*V*_s_=5 mV, *I*_stab_=1 nA and *B*=0.2 T). The magnetic tip is sensitive to the out-of-plane magnetization component. (**c**) Time trace of the Ho atom from **b** shown in a more narrow Δ*Z* range. (**d**) Thirty-nine time traces on 13 different Ho atoms (colour gradient from yellow to orange) recorded with spin-polarized tips and various parameters *I*_stab_ (0.5 to 50 nA), *V*_s_ (3 to 10 mV), *B* (−0.2 to 0.2 T) and recording times *t* (200 to 1,600 s) (see Supplementary Note 3 for a list of parameter combinations). The telegraph noise of the Fe_3_-cluster (blue) from **b** serves as a reference.

As XMCD measurements clearly indicate the presence of a magnetic moment of Ho on Pt(111)^13^, the lack of ISTS signal questions if the tunnelling electrons sufficiently exchange couple with the 4*f*-derived magnetic moment of the Ho atom^16^,^17^. Another approach to probe this is to employ spin-resolved tunnelling, which is sensitive to the spin-polarization in the vacuum^18^. To perform such measurements, we created a magnetic tip by intentionally picking up Fe atoms from the substrate to the tip, until spin-sensitivity was achieved. The spin-sensitivity to the out-of-plane component of the magnetization was quantified by recording the magnetic telegraph noise of the tip height Δ*Z* on an artificially constructed array of three Fe atoms (Fe_3_-array, Fig. 2a,b). The magnetization of this array switches between two degenerate ground states with opposite out-of-plane magnetization components (red and blue in Fig. 2b) similar to Fe_5_ on Cu(111)^5^, as proven by an increase in the asymmetry of the residence times in the two states with *B*. The observed telegraph noise has a strength of Δ*Z*≈15 pm on a noise level of 0.3 pm (r.m.s., time averaging 20 ms).

Using the Fe_3_ as a calibration, we employ tips which showed the above-mentioned contrast to probe individual Ho atoms (Fig. 2b,c). Time traces with calibrated tips and various combinations of the parameters *I*_stab_ (0.5 to 50 nA), *V*_s_ (3 to 10 mV), *B* (−0.2 to 0.2 T) and recording times *t* (200 to 1,600 s), which include the choices of parameters of Miyamachi *et al.*^8^, have been recorded on 13 different Ho atoms (see Supplementary Table 1 in Supplementary Note 3 for the list of parameter combinations). The 39 time traces (Fig. 2d) do not show any indication for the magnetic telegraph noise that has been reported in ref. 8, within a noise level specified above. Recent measurements using XMCD reported a mixture of *J*_*z*_=±6 with an almost quenched expectation value along the surface normal as the magnetic ground state of Ho on Pt(111)^13^. While this would explain the observed absence of telegraph noise in our magnetic measurements in contrast to Miyamachi *et al.*^8^, the corresponding spin-Hamiltonian still enables spin-excitations at a few meV energy, which we do not observe. Therefore, the lack of inelastic and spin-polarized signal from Ho questions if tunnelling-based methods are sensitive enough to interrogate the magnetism of 4*f* single-atom moments. We discuss the latter point in more detail later, by comparing the experimental observations with the calculated electronic structure.

### Study of the magnetic interaction between Ho and Fe atoms

**Figure 3:**
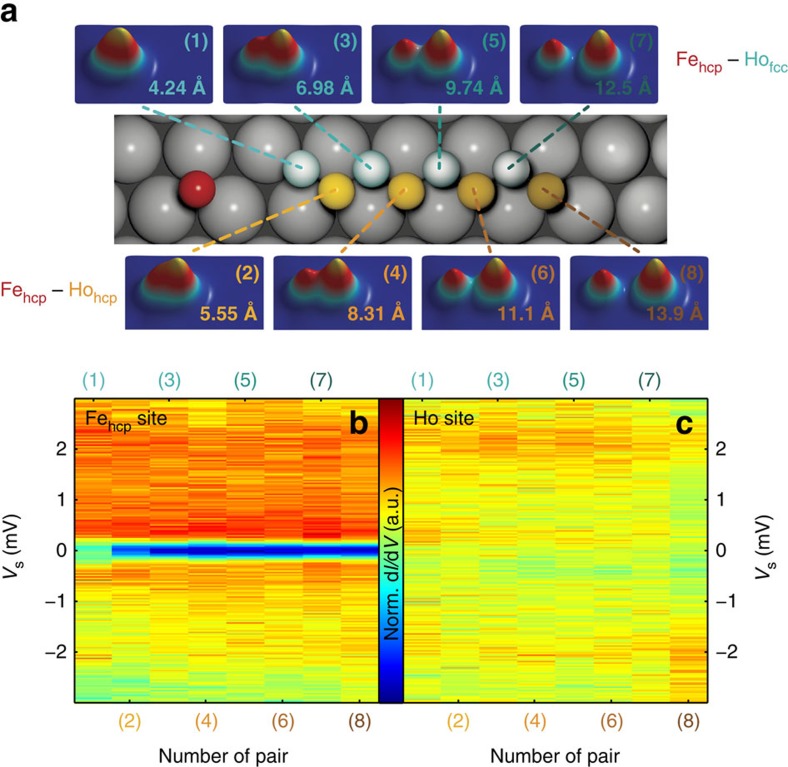
Distance dependency of the magnetic interactions in an Fe–Ho pair. (**a**) Constant-current images [(1–8)] of different Fe_hcp_–Ho atom pairs with indicated distances and related ball-models in between. (**b**) Spectra on the Fe_hcp_ atom and (**c**) the Ho atom for each pair with the 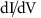 signal represented by the given colour scale. The spectra have been normalized to a substrate spectrum, divided by their mean value and the colour scaling for Fe and Ho is the same (*I*_stab_=3 nA, *V*_stab_=10 mV and *V*_mod_=40 μV).

It is well-known that magnetic order of bulk 4*f* materials is mediated by indirect exchange interactions via exchange of the 4*f* electrons with 5*d* and 6*s* itinerant electrons^11^. At the single-atom limit, it is interesting to ask if the exchange fields produced by single Ho atoms on surfaces behave similar to the bulk. Therefore, we use an Fe atom in close proximity to Ho and monitor the changes of the magnetic excitation of Fe, to probe the indirect exchange field produced by Ho. We have shown in an earlier publication that the Pt(111) substrate mediates an RKKY interaction between two Co atoms adsorbed on the surface with a strength on the order of hundreds of μeV for pair separations in the range of 10 Å (ref. 19). Using atom manipulation, we have built several pairs containing one fixed Fe_hcp_ atom and a Ho atom that was moved to different lattice sites (both fcc and hcp), resulting in various distances between the Fe_hcp_ and the Ho (Fig. 3a). For each pair, we performed ISTS on both atoms (Fig. 3b,c). Remarkably, we observe the following two points: first, the spectra of the coupled Ho atoms do not change regardless of separation or applied magnetic field (Fig. 3 and Supplementary Note 4), and second the spectra of the coupled Fe_hcp_ atom also do not change for separations larger than 5.55 Å (Fig. 3). It was previously shown that a signature of exchange fields between a pair of atoms can be detected as a perturbation to the spin excitation^20^. The lack of any significant changes to the Fe excitations indicate that the indirect exchange field produced by Ho is negligible at our measurement temperature. Only for the two closest pairs (*d*=4.24, 5.55 Å) we observe a change in the measurement. There is a drop of the excitation intensity, but the excitation energy remains constant. It is important to note that we further checked the *B*-dependent ISTS measurements up to 7 T on the closest pair in Supplementary Figure 6 in Supplementary Note 4, which show that the evolution of the spin-excitation of the Fe in this pair is identical to that of an isolated Fe atom. The intensity drop is therefore probably an effect due to the modified vacuum density of states rather than due to a magnetic interaction. Taking into account the narrow linewidth of the spin-excitation step in the Fe_hcp_ spectrum, we can show that the method has a sensitivity of 50 μeV (see Supplementary Figure 7 in Supplementary Note 5). We can thus conclude that the interaction between Fe and Ho in the RKKY regime is smaller than this limit.

### Electronic and magnetic structure calculations

**Figure 4:**
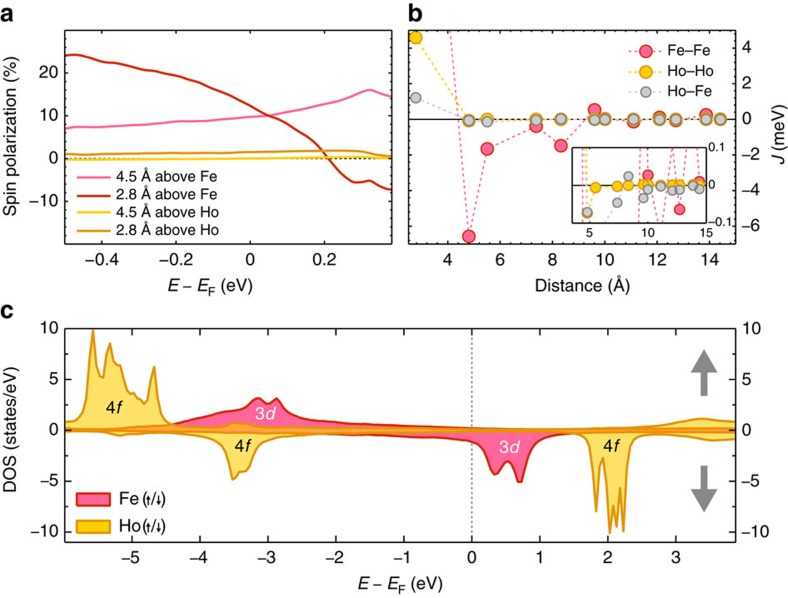
Electronic structure and magnetic calculations of Ho and Fe. (**a**) Spin-polarization in vacuum above Fe_fcc_ (red) and Ho_fcc_ (orange) atoms for two selected tip-atom distances, from DFT calculations. (**b**) Magnetic coupling parameters for Fe–Fe, Fe–Ho and Ho–Ho pairs on hcp sites, obtained from the magnetic force theorem. The inset shows a more narrow window for the coupling strength *J* for a better view on the smallest values. (**c**) Local density of states (LDOS) for isolated Fe_fcc_ (red) and Ho_fcc_ (orange) atoms, showing the location of the 3*d* and 4*f* states with respect to the Fermi energy of platinum. Positive or negative values indicate majority or minority spins, which is also indicated by the grey up or down arrows.

To support these experimental findings, we performed DFT calculations for isolated atoms and pairs using the Korringa–Kohn–Rostoker Green function method^21^. Both Fe_fcc_ and Fe_hcp_ were considered, and spin-orbit coupling as well as an local density approximation (LDA)+U correction for the 4*f* states of Ho were included. Further methodological details are given in Supplementary Note 6, and Supplementary Table 2 in Supplementary Note 7 additionally summarizes the ground state properties of both adatom types. In Fig. 4a, we show the spin-polarization, *P*(*E*)=100(*N*_↑_(*E*)−*N*_↓_(*E*))/(*N*_↑_(*E*)+*N*_↓_(*E*)) with *N*_↑,↓_(*E*) as the number of spin-up or spin-down electrons at the specified energy *E*, in the vacuum at two different heights from an Fe_fcc_ or Ho_fcc_ atom. For Fe, a substantial spin-polarization of ≈10% is obtained for *d*=4.5 Å, increasing up to 30% for *d*=2.8 Å. This results from the spin-polarized 3*d* states of Fe in this energy window. Conversely, the spin-polarization of Ho is below 1%, as the Ho 4*f* density of states is low and not spin-polarized in this energy window. As it is well-known that magnetic atoms can induce a large moment in surrounding Pt atoms^3^,^14^, we calculated the net moment induced in Pt by Fe_fcc_ and Ho_fcc_ independently. For Fe, the induced spin moment is 0.72 *μ*_B_ (in Khajetoorians *et al.*^14^ a larger cluster was used, leading to an induced spin moment of 1.02 *μ*_B_ (see Supplementary Note 6 for details)). This points to a strong magnetic coupling between the Fe 3*d* orbitals and the Pt 5*d* and 6*s* states. For Ho, we get an induced moment of 0.05 *μ*_B_, reinforcing the weak hybridization picture for the 4*f* states.

Figure 4b illustrates the calculated distance-dependent indirect exchange interaction between three different types of hcp–hcp pairs, namely Fe–Fe, Fe–Ho and Ho–Ho. The magnetic coupling of Fe–Ho or Ho–Ho pairs is nearly 100 times smaller than that of the corresponding Fe–Fe pair at the same distance. These results corroborate the experimental findings in Fig. 3, in which there is no evidence of indirect exchange interactions produced by a Ho atom. The root of the weak exchange field produced by Ho can be illustrated by investigating the electronic structure of the Ho atom in comparison to the Fe atom (Fig. 4c). The minority Fe 3*d* orbitals are relatively close to the Fermi energy *E*_F_, while the Ho 4*f* orbitals were pushed away by the LDA+U correction. Overall, the 4*f* states of Ho appear sharper than the 3*d* states of Fe located at similar energies, suggesting a weaker hybridization for Ho–Pt than for Fe–Pt. The magnetic coupling of a dimer is affected by virtual transitions between the 3*d* or 4*f* orbitals of the different atoms, which are sensitive to both the position of the peaks with respect to *E*_F_ and the strength of the hybridization (see Supplementary Figure 8 in Supplementary Note 6 to find a graph showing the dependency of the coupling strength to the U correction in the LDA+U calculation). This results in a much weaker Fe–Ho coupling as compared with the Fe–Fe coupling, which is below the experimental sensitivity. Likewise, the tunnelling conductance is affected by the same quantities in a similar way (see Supplementary Note 8). This implies that the inelastic excitation intensity for Ho is very weak in comparison to Fe, that is, the coupling of tunnelling electrons to the localized 4*f* levels is less probable as compared with the hybridized 3*d* levels, suggesting why there is an absence of inelastic signal in ISTS. The 4*f* hybridization with its electronic environment is overestimated due to insufficient spatial localization of the 4*f* orbitals. This may be better described by a LDA+DMFT type approach, which may also better describe the full multiplet structure of Ho/Pt(111)^22^,^23^.

## Discussion

In summary, our investigations show that the 4*f* orbitals of a Ho atom adsorbed on Pt(111) are very well-isolated from the surrounding electronic and magnetic environment. This has profound implications on atomic-scale magnetic technologies based on rare-earth magnets, as conventional wisdom suggests that magnetic order emerges in rare-earth compounds via coupling pathways between the 4*f* orbitals and itinerant electrons. Our results show that these exchange paths are altered if a single rare-earth atom is adsorbed on a surface, most likely due to the change in coordination and symmetry of the atom compared with the bulk. With regards to the work of Miyamachi *et al.*^8^, the absence of a magnetic and inelastic signal observed here only allows us to speculate about the discrepancy between recent measurements and if the described theoretical proposal was realized, as neither the proper values of the spin-resolved differential conductance, the inelastic intensity nor the sensitivity limits were given in ref. 8. Assuming we have similar detection limits, the observed discrepancy evokes the question, whether the Ho adsorbates investigated here and by Miyamachi *et al.*^8^ could be of different chemical composition. In addition to clean Ho atoms, we observed adsorbates with a different apparent height which we attribute to hydrogen-contaminated Ho. Moreover, the Pt(111) surface contains residual defects, which are often located close to Ho atoms. Hydrogen or other contaminations of adatoms can either strongly change their magnetism^24^ or lead to conformational changes^25^ that can be confused with magnetic excitations in ISTS or magnetic switching in spin-polarized measurements. Before our measurements, we therefore always removed hydrogen from the surface and the adatoms and manipulated the adatom to a clean spot of Pt(111) to exclude such effects. The absence of telegraph noise rules out a ground state of *J*_*z*_=8 for the clean Ho atoms we investigate, in contrast to ref. 8, and corroborates a *J*_*z*_=6 ground state as found by XMCD measurements by Donati *et al.*^13^. However, we do not observe the ISTS signal expected from any of the anisotropy values for the spin-Hamiltonian in refs 8, 13. Our investigation therefore rather questions if tunnelling-based methods can probe 4*f*-derived magnetic atoms, due to the weak interaction and strong localization of the 4*f* orbitals, and if Ho/Pt(111) is the proper system to experimentally investigate the theoretical proposal of Miyamachi *et al.*^8^.

## Methods

### STM/STS measurements

The experiments were performed in a home-built STM facility^26^ at a temperature of *T*=0.3 K in a magnetic field *B* applied perpendicular to the sample surface. We used flashed W tips with a nominal coating of 50 monolayers of Cr for spin-polarized measurements^4^. Magnetic contrast was achieved by picking up a number of Fe atoms, whereas we used a combination of voltage-pulsing and tip-dipping for inelastic ISTS measurements to get feature-less tips within the measured energy range. Constant-current images were recorded at a tunnelling current *I*_stab_ with a bias voltage *V*_s_ applied to the sample. ISTS was performed by adding a modulation voltage *V*_mod_ (r.m.s.) of frequency *f*_mod_ to *V*_s_, stabilizing the tip at *I*_stab_ and *V*_stab_, switching the feedback off, ramping the bias voltage and recording the d*I*/d*V* signal using a lock-in amplifier with time constant 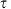 and sensitivity *V*_sens_. The Pt(111)-crystal was cleaned *in situ* as described in ref. 14. Ho atoms were evaporated onto the surface from a Knudsen-cell at a temperature of 900 °C for 20 s and a base pressure of 9 × 10^−11^ mbar. Afterwards, single Fe atoms were co-deposited onto the same sample using an e-beam evaporator with an Fe rod. During deposition, the sample temperature did not exceed 20 K. The result is a statistical distribution of Ho and Fe atoms on the Pt(111) surface (Supplementary Note 1 and Supplementary Figure 1).

### Electronic and magnetic structure calculations

We used the Korringa–Kohn–Rostoker Greeen function method^21^ for the DFT calculations of single Fe and Ho atoms both on hcp and fcc lattice sites. Spin-orbit coupling was included and a LDA+U correction was applied for the 4*f* states of Ho. A slab of 22 Pt layers stacked in the (111) direction augmented by two vacuum regions was used to define the undisturbed Pt(111) surface. Both the Fe and Ho atoms were relaxed vertically towards the surface by 20%. To correctly take into account for the hybridization of *d* and *f* states with itinerant surface electrons, the Anderson impurity model in the mean-field approximation was used. For more details about the performed calculations, we would like to refer the reader to the provided Supplementary Notes 6–8.

## Additional information

**How to cite this article:** Steinbrecher, M. *et al.* Absence of spin-signature of a single Ho adatom as probed by spin-sensitive tunnelling. *Nat. Commun.* 7:10454 doi: 10.1038/ncomms10454 (2016).

## Supplementary Material

Supplementary Information Supplementary Figure 1-8, Supplementary Table 1-2, Supplementary Notes 1-8 and Supplementary References
