# Supplementary material for: Absence of a spin-signature from a single Ho adatom as probed by spin-sensitive tunneling
Source: Nat Commun. 2016 Feb 3;7:10454. doi: 10.1038/ncomms10454 (PMC4742789; doi:10.1038/ncomms10454)
Supplement: Supplementary Information — Supplementary Figure 1-8, Supplementary Table 1-2, Supplementary Notes 1-8 and Supplementary References [file ncomms10454-s1.pdf]

SUPPLEMENTARY INFORMATION

Supplementary Note 1 | Overview of the sample system

We co-deposit iron (Fe) and holmium (Ho) atoms onto the platinum (Pt) (111) surface and observe a stochastic distribution of single atoms as shown in Supplementary Figure 1. Here, Fe atoms which have a smaller apparent height appear as green dots whereas the Ho atoms which have a larger apparent height appear as red dots. The dark spots are defects of the Pt(111) surface. The apparent heights of both, Fe and Ho atoms, show a bias dependence which has been measured with a fixed current of $I_{\mathrm{stab}} = 0.5\,\mathrm{nA}$ as shown in Supplementary Figure 2. The increase in height, which is most dominant for positive bias, can be probably attributed to a bound state formed with the unoccupied surface state of Pt(111) located at $\approx 0.3\,\mathrm{eV}$[1]. Averaged over fcc and hcp adsorption sites, the Fe and Ho atoms show a maximum height of $\approx 145\,\mathrm{pm}$ and $\approx 200\,\mathrm{pm}$, respectively, at $V_s = 1.5\,\mathrm{V}$.

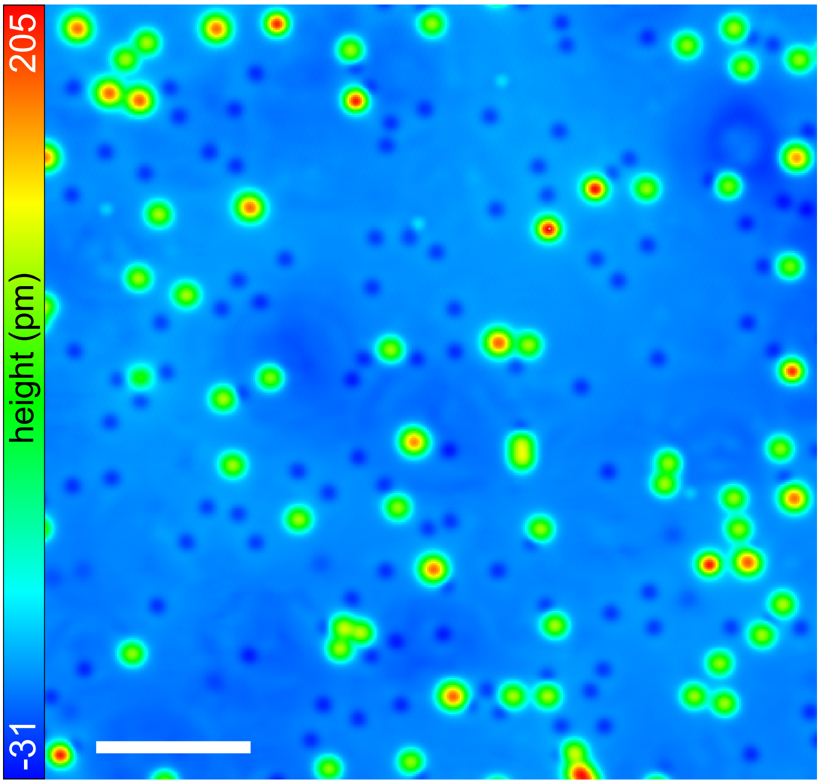

Supplementary Figure 1 | Topographic overview of Ho & Fe adatoms on Pt(111). The $25 \times 25\,\mathrm{nm}^2$ large image was recorded in constant-current mode and shows a statistical distribution of deposited Ho (red) and Fe atoms (green) on a Pt(111) surface. The dark spots can be assigned to defects on the platinum surface. Tunneling parameters were $V_\mathrm{s} = 50\,\mathrm{mV}$ and $I_\mathrm{stab} = 0.5\,\mathrm{nA}$. The white scale bar has a width of $5\,\mathrm{nm}$ and the color scale represents the measured apparent height in a range from -31 to $205\,\mathrm{pm}$.

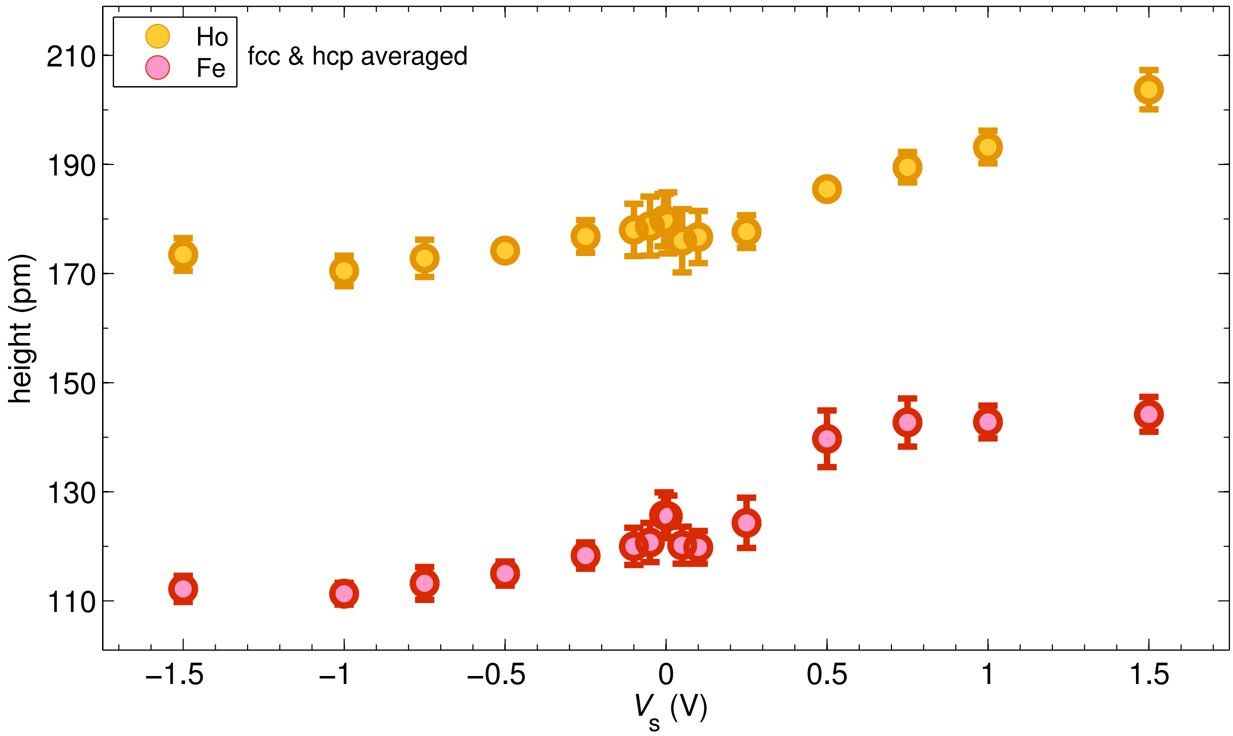

Supplementary Figure 2 | Bias dependent apparent heights. Apparent heights of Fe (red) and Ho (orange) atoms as a function of the applied bias voltage V_s at a current of $I_{\mathrm{stab}} = 0.5\,\mathrm{nA}$. The measurements have been averaged over fcc and hcp adsorption sites and the error bars are defined by twice the standard deviation of the measurements on various atoms.

Supplementary Note 2 | Additional ISTS measurements

In Figure 1 of the main manuscript we have shown 1st derivative ISTS measurements on single Fe and Ho atoms on fcc and hcp lattice sites. In order to compare to Miyamachi *et al.*[2] where the STS spectra were given as 2nd derivative of the tunneling current, we additionally performed similar measurements. For these, a second lock-in amplifier with $V_{\mathrm{sens,2}}$ and time constant τ_2 was used to detect the signal of the 2nd harmonic separately from the 1st harmonic. Again, V_{s} was modulated with a voltage V_{mod} (rms) of frequency f_{mod}. The ${}^{\mathrm{d}^2I}/_{\mathrm{d}V^2}$ signal is calculated from the lock-in output voltage $V_{\mathrm{lock-in}}$ via ${}^{\mathrm{d}^2I}/_{\mathrm{d}V^2} = V_{\mathrm{sens,2}} \cdot V_{\mathrm{lock-in}}/(10\,\mathrm{V} \cdot V_{\mathrm{mod}}^2 \cdot \mathrm{gain})$, where the gain of the preamp is given in volts per ampere. In Supplementary Figure 3 we show corresponding spectra using a moderate tunneling current and modulation voltage taken with the same microtip above Fe and Ho atoms. For $\mathrm{Fe_{fcc}}$ and $\mathrm{Fe_{hcp}}$ the spectra show peaks occuring at the spin-excitation energies that have been previously extracted from the 1st derivative spectra[3] (dashed lines). However, no indication of an inelastic excitation is observed on the Ho atom, in particular not at the previoulsy reported energies[2] (dashed lines).

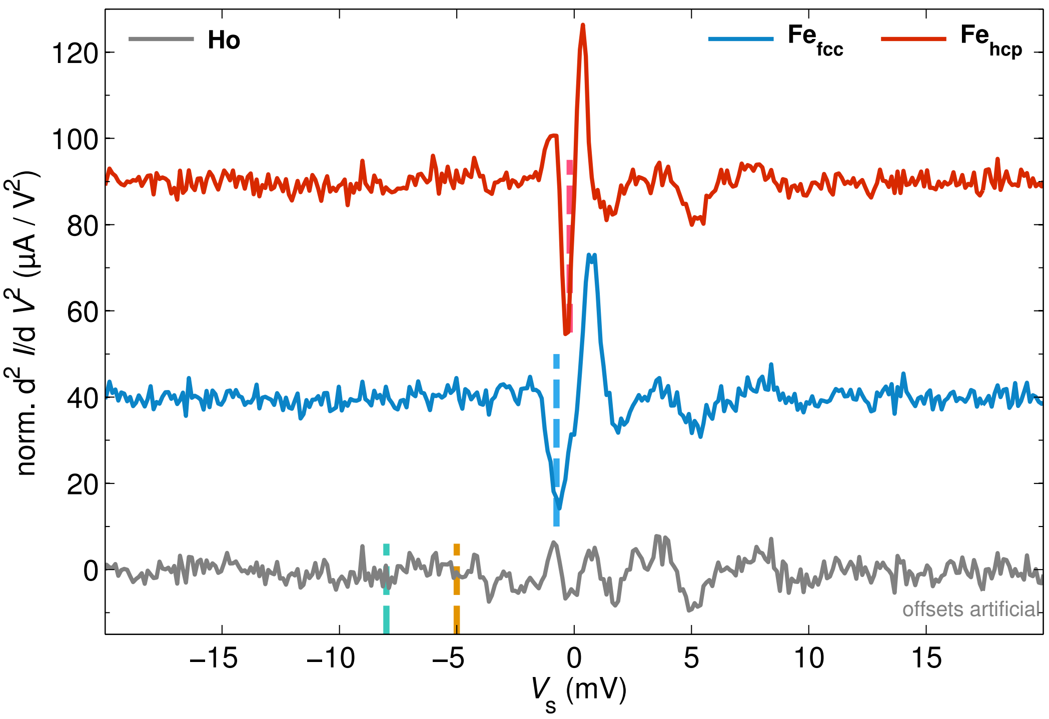

Supplementary Figure 3 | **2nd derivative ISTS measurements.** Normalized (substrate subtracted) 2nd derivative ($^{\mathrm{d}^2 I}/_{\mathrm{d}V^2}$) excitation spectra on Fe and Ho atoms ($V_{\text{stab}} = 25\,\text{mV}$, $I_{\text{stab}} = 14\,\text{nA}$, $V_{\text{mod}} = 0.25\,\text{mV}$, $f_{\text{mod}} = 4.142\,\text{kHz}$, gain $= 0.1\,\text{V}\,\text{nA}^{-1}$, $V_{\text{sens,2}} = 1\,\text{mV}$, $\tau_2 = 10\,\text{ms}$). The Fe spectra are artificially offset and the dashed lines indicate previously reported spin-excitation energies[2,3].

Measurements with parameters very similar to Miyamachi *et al.*[2] and Balashov *et al.*[4] are shown in Supplementary Figs. 4 and 5. Note, that the modulation voltage V_{mod} slightly differs from that used in Refs. 2 and 4 due to the damping of a mounted low-pass filter in the high frequency range ($f_{\text{mod}} = 16.43\,\text{kHz}$). In both figures, the spectra have been taken with the same microtip and the raw spectra are plotted in the top (a) and the normalized (substrate subtracted) spectra in the bottom (b) of the figure. Due to the large modulation voltage there is a shift of the measured Fe spin-excitations to higher energies with respect to the real excitation energies (dashed lines) as well as a loss in intensity. It is important to note, that we were not able to produce tips which show a flat substrate signal in the measured energy window. Instead, even the flattest substrate spectra show variations due to tip-related features (see black curves in Supplementary Figs. 4(a) and 5(a)). These features are not fully removed from the spectra measured on the atoms by subtracting a substrate spectrum measured with the same tip (Supplementary Figs. 4(b) and 5(b)). This effect leads to variations in the spectra measured on the atoms which, in the best case, amounts to $\pm 2\,\mu\text{A}\,\text{V}^{-2}$. Nevertheless, by comparing spectra measured on Ho atoms using different tips, we can conclude that there is no reproducible signature of a spin-excitation at the previously reported energies (dashed lines[2]) on top of the noise level of $\pm 0.5\,\mu\text{A}\,\text{V}^{-2}$.

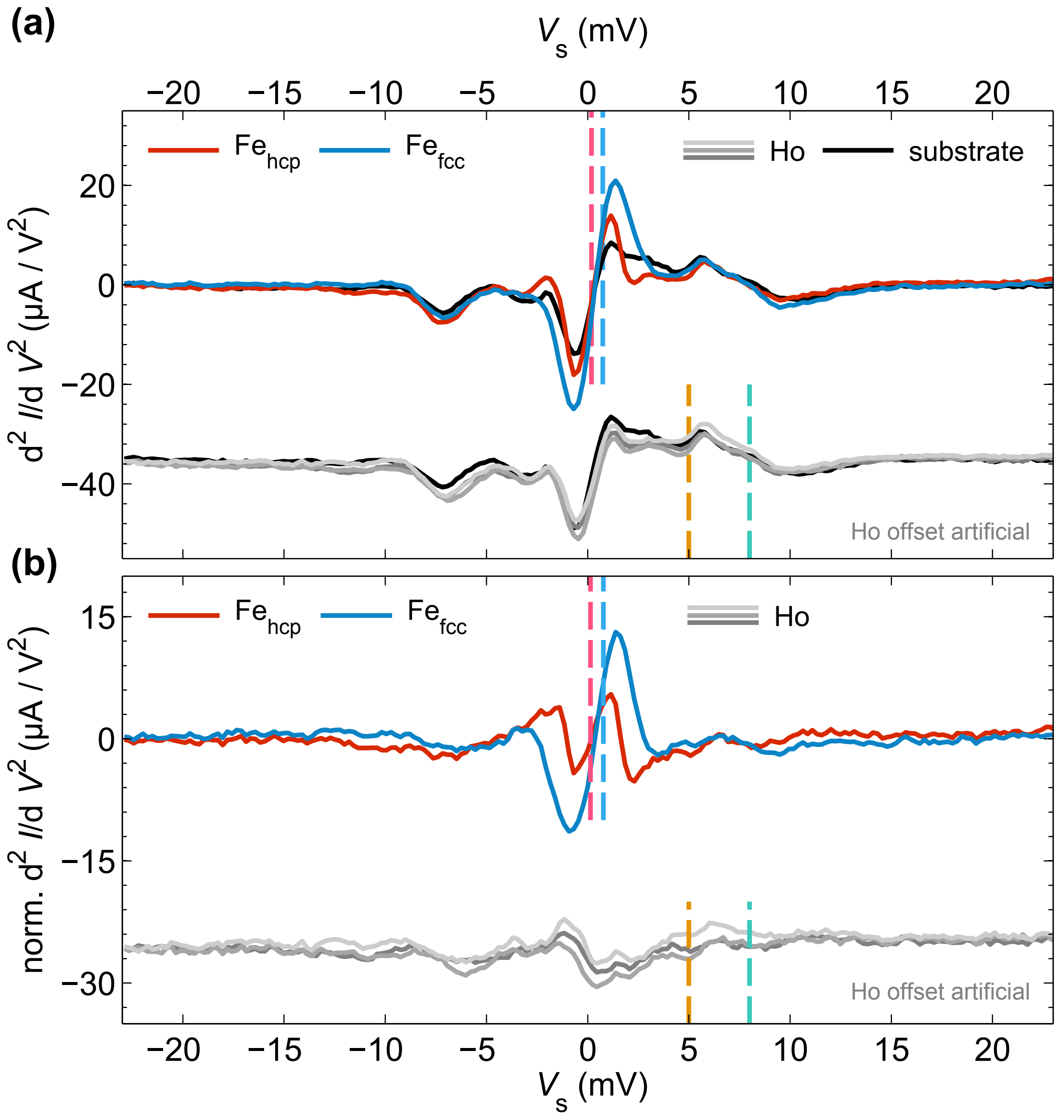

Supplementary Figure 4 | 2nd derivative ISTS measurements with different parameters #1. Raw **(a)** and normalized **(b)** $^{d^2I}/_{dV^2}$-spectra with different settings on Fe and Ho atoms ($V_{stab} = -23\,mV$, $I_{stab} = 29\,nA$, $V_{mod} = 0.92\,mV$, $f_{mod} = 16.43\,kHz$, gain $= 0.01\,V\,nA^{-1}$, $V_{sens,2} = 1\,mV$, $\tau_2 = 30\,ms$). The Ho spectra are artificially offset and the dashed lines indicate previously reported spin-excitation energies[2,3].

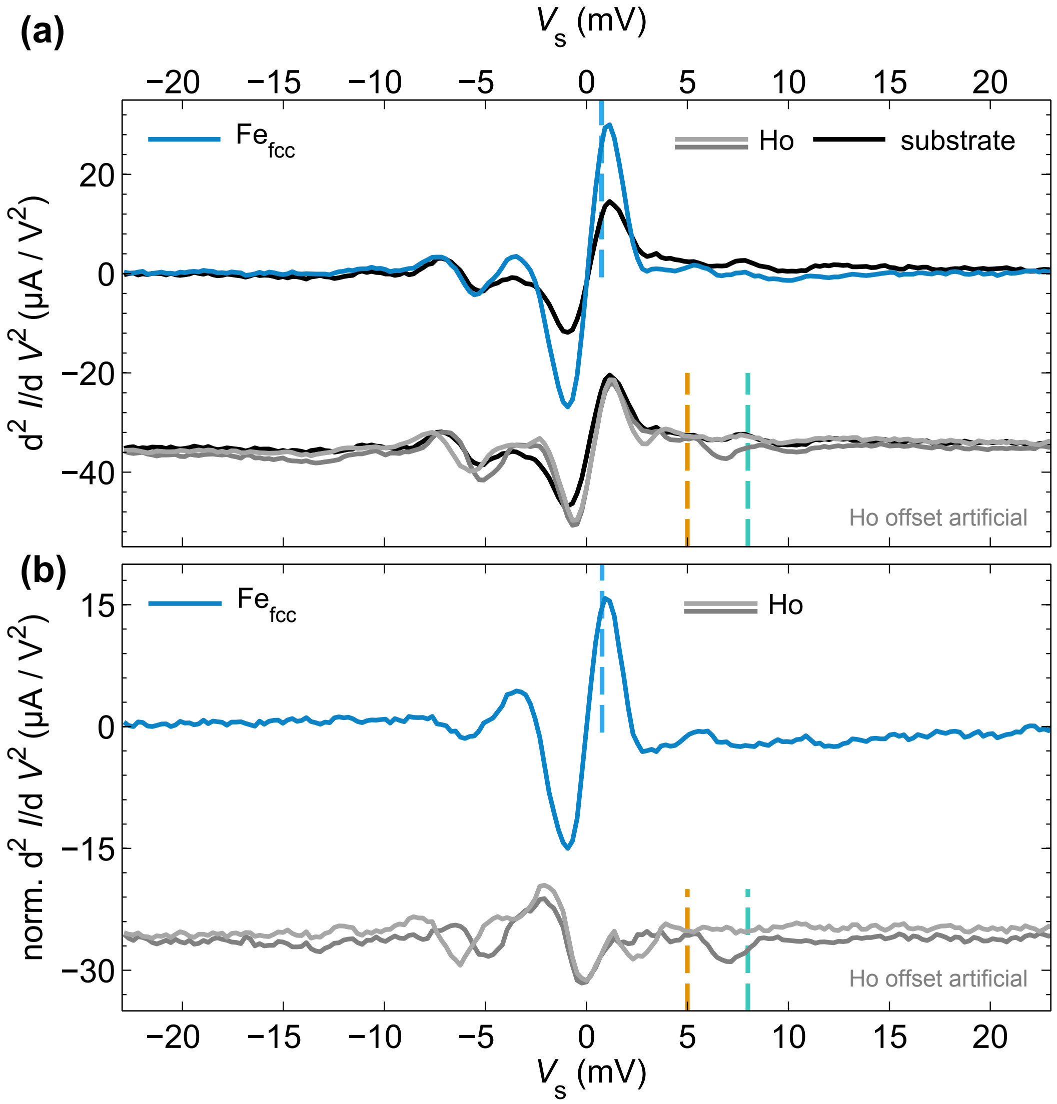

Supplementary Figure 5 | 2nd **derivative ISTS measurements with different parameters #2.** Raw (**a**) and normalized (**b**) $^{\mathrm{d}^2 I}/_{\mathrm{d}V^2}$-spectra with different settings on Fe and Ho atoms ($V_{\mathrm{stab}} = 23\,\mathrm{mV}$, $I_{\mathrm{stab}} = 29\,\mathrm{nA}$, $V_{\mathrm{mod}} = 0.92\,\mathrm{mV}$, $f_{\mathrm{mod}} = 16.43\,\mathrm{kHz}$, gain $= 0.01\,\mathrm{V\,nA^{-1}}$, $V_{\mathrm{sens},2} = 1\,\mathrm{mV}$, $\tau_2 = 30\,\mathrm{ms}$). The Ho spectra are artificially offset and the dashed lines indicate previously reported spin-excitation energies[2,3].

Supplementary Note 3 | Parameters of the spin-polarized measurements

For the 39 time traces of Figure 2(d) in the main manuscript, a variety of different combinations of parameters have been used, which are given in Supplementary Table 1. The largest achieved spin-polarization resulted in a strength of the telegraph noise measured above the Fe_3 of $\Delta Z \approx 35\,\mathrm{pm}$.

Supplementary Table 1 | Parameters used for time-dependent spin-resolved measurements. Combinations of the parameters stabilization current I_{stab}, sample bias V_{s}, magnetic field B and recording time t of all 39 time traces shown in Figure 2(d) of the main manuscript.

I_{stab} (nA)	V_{s} (mV)	B (T)	t (s)
0.5	3	0.1	985
0.5	5	0.1	770
0.5	10	0.1	720
1	5	-0.2	188
1	5	-0.2	255
1	5	0	575
1	5	0	580
1	5	0	580
1	5	0	760
1	5	0.1	267
1	5	0.1	371
1	5	0.1	570
1	5	0.1	570
1	5	0.1	580
1	5	0.1	580
1	5	0.1	1150
1	5	0.2	271
1	5	0.2	322
1	7.5	0	214
1	10	-0.2	230
1	10	-0.2	232
1	10	0	562
1	10	0	575
1	10	0	590
1	10	0	600
1	10	0	605
1	10	0.1	348
1	10	0.1	563
1	10	0.1	580
1	10	0.1	590
1	10	0.1	620
1	10	0.1	650
1	10	0.2	324
1	10	0.2	390
5	5	0.1	1600
10	5	0.1	1140
20	5	0.1	1110
40	5	0.1	1175
50	5	0.1	1230

Supplementary Note 4 | B**-field dependent ISTS of the** $4.24\,\text{Å}$ **Fe-Ho pair**

In order to study the influence of the Ho atom on the magnetism of Fe_{hcp}, we carried out ISTS investigations of the closest pair in Figure 3 with $4.24\,\text{Å}$ distance in a magnetic field up to $7\,\text{T}$. For comparison, the same experiment was done on a single Fe_{hcp} atom and a single Ho_{fcc} atom. The results are shown in Supplementary Figure 6 with the normalized inelastic spectra in Supplementary Figure 6(a-d) and a representation as a color plot in Supplementary Figure 6(e-h). For the single Fe_{hcp} atom the results of[3] are reproduced, i.e. a zero field spin-excitation at $0.19\,\text{meV}$ followed by a decrease for small magnetic fields and finally a linear increase above $4\,\text{T}$. The same behaviour with identical energetic positions of the excitation is observed for the Fe_{hcp} atom in the pair. However, the excitation intensity is slightly reduced. Note, that this change in intensity is independent of the magnetic field. This indicates that the change in intensity is most likely not due to magnetic coupling, but due to an effect of the Ho atom on the measured vacuum density of states.

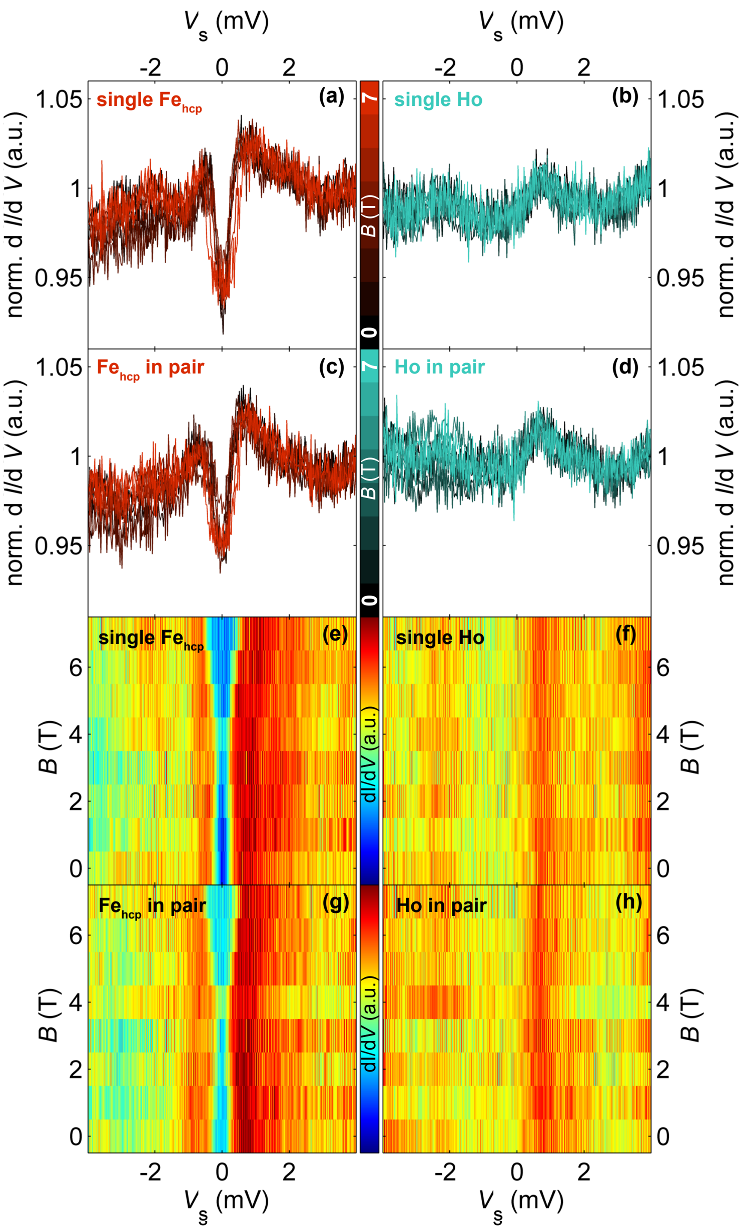

Supplementary Figure 6 | ISTS in B_z-field of Fe and Ho atoms, single and in mixed pairs. Magnetic field dependent ISTS measurements up to $B = 7\,\mathrm{T}$ for the $\mathrm{Fe_{hcp}}$-$\mathrm{Ho_{fcc}}$ pair with $4.24\,\mathrm{nm}$ distance ($I_{\mathrm{stab}} = 3\,\mathrm{nA}$, $V_{\mathrm{stab}} = 6\,\mathrm{mV}$ and $V_{\mathrm{mod}} = 40\,\mathrm{\mu V}$). (**a**) & (**b**) as well as (**e**) & (**f**) represent reference measurements on an individual $\mathrm{Fe_{hcp}}$ and Ho atom. (**c**) & (**d**) as well as (**g**) & (**h**) show the measurements on the atoms within the pair. The color plot intensity is derived by dividing each spectrum at a given magnetic field by its mean value. The higher and lower limits of the color scales are the same for all plots.

Supplementary Note 5 | Detection limit of magnetic interactions

In the main manuscript, the energetic position of the Fe_{hcp} spin-excitation is used as a sensor for the magnetic coupling to a nearby Ho atom. In order to estimate the sensitivity of this method we have analyzed the linewidth of the Fe_{hcp} spin-excitation. In Supplementary Figure 7 the analytical derivative of the Fe_{hcp} spectrum from Figure 1 of the main manuscript is plotted. By fitting the peak of the excitation to a gaussian we find a linewidth of $84 \pm 10\,\mu\mathrm{eV}$. Assuming that the shift and broadening of the excitation peak are to first order proportional to the magnetic coupling strength, we estimate that we can still detect a shift of $\approx 50\,\mu\mathrm{eV}$ of the excitation (Supplementary Figure 7), which quantifies the sensitivity of this method.

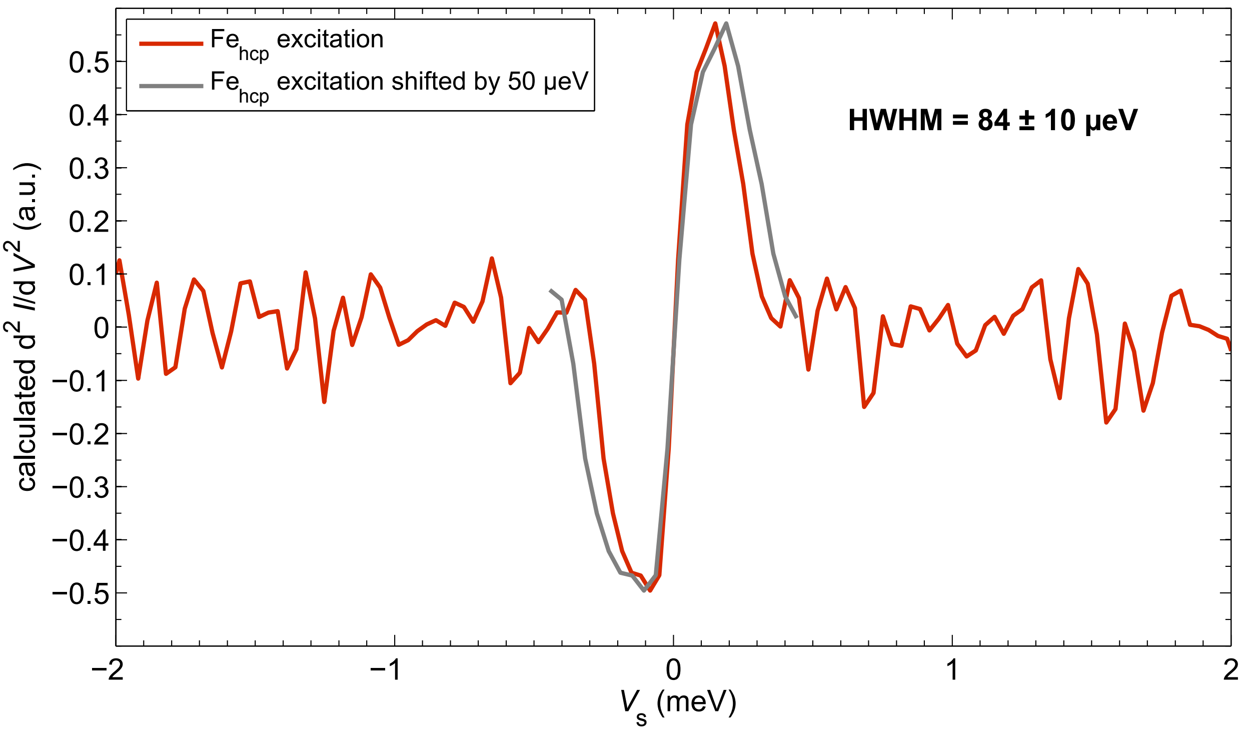

Supplementary Figure 7 | Energy resolution of ISTS peaks. Numerically differentiated Fe_{hcp} spectrum (red) from Figure 1. By fitting the peak of the spin-excitation, a linewidth of $84 \pm 10\,\mu\mathrm{eV}$ can be extracted. If the excitation is shifted by $50\,\mu\mathrm{eV}$, the two spectra can still clearly be distinguished.

Supplementary Note 6 | DFT calculations

DFT calculations were performed with the KKR–GF in a real-space approach within the atomic sphere approximation and including spin–orbit coupling[5]. The Local Spin Density Approximation (LSDA), as parametrized by Vosko, Wilk and Nusair, was adopted[6]. A slab of 22 Pt layers stacked in the (111) direction augmented by two vacuum regions was used to define the undisturbed Pt(111) surface, using the experimental lattice constant ($a = 3.92$ Å) and without surface relaxations (they are typically $\pm 1\%$[7]). From this surface, a real space cluster is cut out surrounding the position to be occupied by the Fe or Ho atoms (fcc: 27, hcp: 29 atoms) and the corresponding dimers (fcc: 110, hcp: 116 atoms). The atoms and dimers are placed in threefold fcc or hcp hollow sites (mixed fcc-hcp dimers cannot be computed within our approach), relaxed vertically 20% towards the surface as in our previous work[3] (0% corresponds to the ideal interlayer separation in bulk, $a/\sqrt{3} = 2.26$ Å).
For Ho, it was found that the preferred vertical distance is actually slightly larger than the bulk interlayer distance[2]. However the electronic properties become quite insensitive to the relaxation once the LDA+U correction is applied, as checked in our calculations. Consequently, we placed Ho at the same vertical distance from the surface as Fe.
The magnetic interactions for the Fe–Fe, Fe–Ho and Ho–Ho dimers were computed using the generalized Lichtenstein formula from Ebert and Mankovsky[8], extended to the LDA+U case. The impact of the LDA+U correction on the magnetic interactions is exemplified in Supplementary Figure 8.
Part of the $4f$ orbitals of Ho in the LSDA are incorrectly located at the Fermi energy (E_F). Therefore, we apply the simplest LDA+U correction, the Dudarev form[9],

$$E_\mathrm{LDA+U} = \frac{U_\mathrm{eff}}{2}\rho_f\big(1 - \rho_f\big) \tag{1}$$

with $U_\mathrm{eff} = 5\,\mathrm{eV}$, and setting up the $4f$ density matrix ρ_f according to Hund's rules. The resulting $4f$ DOS (Figure 4(c) of the main text) agrees well with the experimental spectra for bulk Ho[10] (occupied $4f$ states below $E_\mathrm{F} - 5\,\mathrm{eV}$, empty $4f$ states above $E_\mathrm{F} + 1.5\,\mathrm{eV}$). As the Dudarev correction does not substantially increase the spatial localization of the $4f$ states, their hybridization with the itinerant electrons is still overestimated.

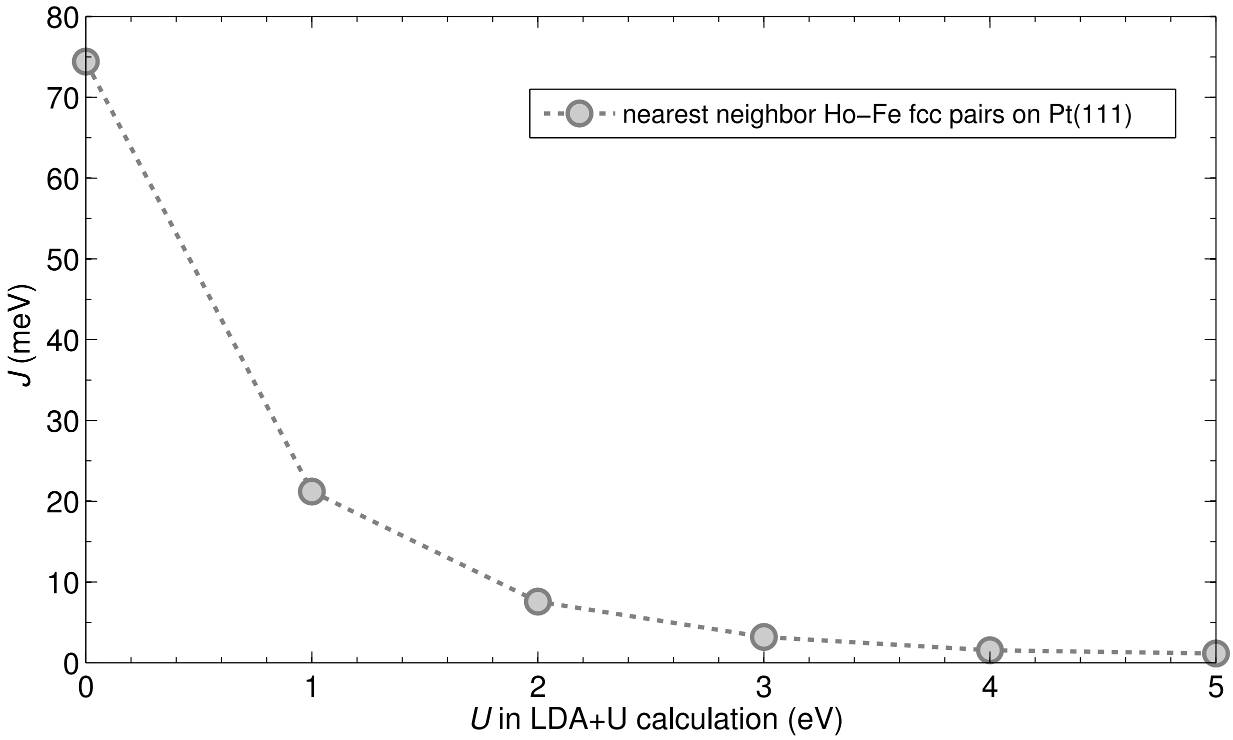

Supplementary Figure 8 | Coupling strength dependency on the parameter U. Dependence of the magnetic coupling of a Ho–Fe dimer on the strength of the U parameter used in the LDA+U correction to the $4f$ states. The large LSDA value for J originates from the presence of the $4f$ states at the Fermi energy. Increasing U pushes the $4f$ states away from the Fermi energy (Figure 4(c) of the main text), resulting in a $1/U$ dependence of the magnetic coupling J.

Supplementary Note 7 | Ground state properties of Fe and Ho adatoms

Supplementary Table 2 summarizes ground state properties of Fe and Ho adatoms on fcc and hcp stacking sites. The $4f$ states of Ho are quite insensitive to the stacking position. The LDA+U ($U_{\text{eff}} = 5\,\text{eV}$) correction brings the $4f$ spin and orbital moments close to the Hund's rules ($2S$ = 4 and L = 6), while the shift of the $4f$ orbitals away from E_{F} reduces the spin moment of the Pt atoms by a factor of four to five. The Fe $3d$ states are more sensitive to the stacking position, in particular the orbital moment changes by $20\,\%$. The induced spin moment created by the Fe adatom also changes by $20\,\%$ between fcc and hcp stacking; is it 15 times larger than the induced spin moment from the LDA+U corrected Ho adatom calculations.

Supplementary Table 2 | Ground state properties of Fe and Ho adatoms on fcc and hcp stacking sites. Calculations were performed including spin-orbit coupling in the LSDA calculations for Fe and Ho (label SOC) and also with the LDA+U correction to the $4f$ states for Ho (label SOC+U, $U_{\text{eff}} = 5\,\text{eV}$). The partial charges, spin and orbital moments (in Bohr magnetons) coming from specific angular momentum contributions in the adatom atomic sphere are labeled in parentheses. The last column reports the total spin moment induced by each adatom on its surroundings.

**Ho**	$Q\,(f)$	$M_{\text{spin}}\,(f)$	$M_{\text{orb}}\,(f)$	$M_{\text{spin}}\,(spd)$	M_{spin} (Pt)
fcc SOC	10.32	3.582	4.985	0.071	0.254
hcp SOC	10.32	3.588	5.006	0.071	0.235
fcc SOC+U	10.14	3.914	5.879	0.067	0.051
hcp SOC+U	10.14	3.913	5.880	0.067	0.051

**Fe**	$Q\,(d)$	$M_{\text{spin}}\,(d)$	$M_{\text{orb}}\,(d)$	$M_{\text{spin}}\,(spf)$	M_{spin} (Pt)
fcc SOC	6.33	3.381	0.171	0.090	0.723
hcp SOC	6.34	3.397	0.140	0.095	0.894

Supplementary Note 8 | Anderson impurity model and scanning tunneling spectroscopy

The hybridization of the $4f$ states with the itinerant electron states is expected to be much smaller than that of the $3d$ electrons[11]. In this section we give a justification in order to show that this reduced hybridization makes coupling to the $4f$ states much harder than to the $3d$ states, both via tunneling electrons or RKKY interactions.

In the Tersoff-Hamann approximation[12], the tunneling conductance is proportional to the density of electronic states of the surface, ρ_s, in the vicinity of the STM tip

$$\frac{\mathrm{d}I}{\mathrm{d}V} \propto \rho_s(E_F + eV; \vec{R}_t) \tag{2}$$

where $\vec{R}_t$ is the tip location, E_F the Fermi energy of the substrate and V the applied bias voltage. The localized d or f orbitals (Fe and Ho, respectively) contribute to the density of states in vacuum through their hybridization with the itinerant surface electrons. This can be qualitatively understood using the Anderson impurity model (in the mean-field approximation):

$$\mathcal{H} = \sum_{\sigma} \big(E_d + U_d\langle n_{d\bar{\sigma}}\rangle\big)\, d^{\dagger}_{\sigma} d_{\sigma} + \sum_{k\sigma} V_d\, \hat{c}^{\dagger}_{k\sigma} d_{\sigma} + \text{h.c.} + \sum_{k\sigma} E_k\, c^{\dagger}_{k\sigma} c_{k\sigma} \quad . \tag{3}$$

The operators $d^{\dagger}_{\sigma}$ and d_{σ} ($\sigma = \uparrow, \downarrow$), create and annihilate electrons in the localized orbital, with energy $E_d + U_d\langle n_{d\bar{\sigma}}\rangle$, $\bar{\sigma} = \downarrow, \uparrow$, which depends on the average occupation of the localized level, $\langle n_{d\bar{\sigma}}\rangle$. The operators $c^{\dagger}_{k\sigma}$ and $c_{k\sigma}$ (k labels the states) create and annihilate electrons in the itinerant

states, with energy E_k. Lastly, V_d controls the hybridization between the localized electrons and the itinerant ones.

The change in the surface DOS arising from the presence of the localized electrons is given by

$$\Delta\rho_s(E;\vec{R}_t) = -\frac{1}{\pi}\,\mathrm{Im}\sum_\sigma C(\vec{R}_t)\,|V_d|^2\,G_{d\sigma}(E) \quad . \tag{4}$$

The complex factor $C(\vec{R}_t)$ contains the information about the decay into vacuum of the electronic states, $|V_d|^2$ is the strength of the hybridization between itinerant and localized electrons, and the information about the localized states is in the local Green's function

$$G_{d\sigma}(E) = \frac{1}{E - E_d - U_d\langle n_{d\bar{\sigma}}\rangle + \mathrm{i}\,\bar{\rho}_s|V_d|^2} \tag{5}$$

from which the DOS of the localized states is derived

$$\rho_d(E) = -\frac{1}{\pi}\,\mathrm{Im}\,G_{d\sigma}(E) = \frac{1}{\pi}\,\frac{\bar{\rho}_s|V_d|^2}{\big(E - E_d - U_d\langle n_{d\bar{\sigma}}\rangle\big)^2 + \big(\bar{\rho}_s|V_d|^2\big)^2} \quad . \tag{6}$$

It consists of two peaks, for spin up and spin down, in qualitative agreement with the full DFT calculations. $|V_d|^2$ can thus be estimated from the width of the peaks of the localized orbitals, and correlated with the tunneling conductance via Supplementary Equations (2) and (4).

The same steps can be followed for two impurities coupled indirectly through the itinerant electrons. The Anderson model now reads (d for Fe $3d$ orbitals and f for Ho $4f$ orbitals)

$$\begin{aligned}\mathcal{H} = \sum_\sigma \big(E_d + U_d\langle n_{d\bar{\sigma}}\rangle\big)\,d^\dagger_\sigma d_\sigma + \sum_{k\sigma} V_d\,\hat{c}^\dagger_{k\sigma} d_\sigma + \text{h.c.} + \sum_\sigma \big(E_f + U_f\langle n_{f\bar{\sigma}}\rangle\big)\,f^\dagger_\sigma f_\sigma \\ + \sum_{k\sigma} V_f\,\hat{c}^\dagger_{k\sigma} f_\sigma + \text{h.c.} + \sum_{k\sigma} E_k\,c^\dagger_{k\sigma} c_{k\sigma} \quad .\end{aligned} \tag{7}$$

and we obtain the local d and f Green's functions as

$$G_{d\sigma}(E) = \frac{g_{d\sigma}(E)}{1 + \bar{\rho}_s^2|V_d|^2|V_f|^2\,g_{d\sigma}(E)\,g_{f\sigma}(E)} \quad , \quad G_{f\sigma}(E) = \frac{g_{f\sigma}(E)}{1 + \bar{\rho}_s^2|V_d|^2|V_f|^2\,g_{d\sigma}(E)\,g_{f\sigma}(E)} \tag{8}$$

where $g_{d\sigma}(E)$ and $g_{f\sigma}(E)$ are the local Green's functions for single impurities, c.f. Supplementary Equation (5).

The change in the energy arising from the indirect coupling between the two impurities can be approximated as (weak coupling)

$$\Delta E_{df} \approx \frac{1}{\pi}\,\mathrm{Im}\int^{E_F}\mathrm{d}E\;\bar{\rho}_s^2|V_d|^2|V_f|^2\sum_\sigma g_{d\sigma}(E)\,g_{f\sigma}(E) \tag{9}$$

The magnetic interaction between the two impurities is defined as the energy difference between the antiferro- and ferromagnetic configurations

$$J_{df} = \Delta E_{df}(\uparrow\downarrow) - \Delta E_{df}(\uparrow\uparrow) \tag{10}$$

Consequently the magnetic coupling for df (Fe–Ho) and dd (Fe–Fe) dimers scales as

$$\frac{J_{df}}{J_{dd}} \propto \frac{|V_f|^2}{|V_d|^2} \ll 1 \tag{11}$$

with a similar scaling expected for the tunneling conductance in vacuum, c.f. Supplementary Equation (4):

$$\frac{\Delta\rho_{sf}(E;\vec{R}_t)}{\Delta\rho_{sd}(E;\vec{R}_t)} \propto \frac{|V_f|^2}{|V_d|^2} \ll 1 \tag{12}$$

The conclusion is, that, given a small hybridization ratio, it is as hard to induce inelastic excitations in a Ho adatom via tunneling electrons, Supplementary Equation (12), as it is to magnetically couple to it via a second Fe adatom, Supplementary Equation (11).

Supplementary References

1. Wiebe, J. *et al.* Unoccupied surface state on Pt(111) revealed by scanning tunneling spectroscopy. *Physical Review B* **72**, 193406 (2005).

2. Miyamachi, T. *et al.* Stabilizing the magnetic moment of single holmium atoms by symmetry. *Nature* **503**, 242–6 (2013).

3. Khajetoorians, A. A. *et al.* Spin excitations of individual fe atoms on pt(111): Impact of the site-dependent giant substrate polarization. *Phys. Rev. Lett.* **111**, 157204 (2013).

4. Balashov, T. *et al.* Dynamic magnetic excitations in 3d and 4f atoms and clusters. *Surface Science* **630**, 331 – 336 (2014).

5. Papanikolau, N., Zeller, R. & Dederichs, P. Conceptual improvements of the kkr method. *Journal of Physics: Condensed Matter* **14**, 2799 (2002).

6. Vosko, S., Wilk, L. & Nusair, M. Accurate spin-dependent electron liquid correlation energies for local spin-density calculations - a critical analysis. *Can. J. Phys.* **58**, 1200–1211 (1980).

7. Blonski, P. & Hafner, J. Density-functional theory of the magnetic anisotropy of nanostructures: an assessment of different approximations. *J. Phys.: Condens. Matter* **21**, 426001 (2009).

8. Ebert, H. & Mankovsky, S. Anisotropic exchange coupling in diluted magnetic semiconductors: Ab initio spin-density functional theory. *Physical Review B* **79**, 045209 (2009).

9. Dudarev, S. L., Botton, G. A., Savrasov, S. Y., Humphreys, C. J. & Sutton, A. P. Electron-energy-loss spectra and the structural stability of nickel oxide: An LSda+u study. *Phys. Rev. B* **57**, 1505–1509 (1998).

10. Lang, J. K., Baer, Y. & Cox, P. A. Study of the 4f and valence band density of states in rare-earth metals. ii. experiment and results. *Journal of Physics F: Metal Physics* **11**, 121 (1981).

11. van der Marel, D. & Sawatzky, G. A. Electron-electron interaction and localization in *d* and *f* transition metals. *Phys. Rev. B* **37**, 10674–10684 (1988).

12. Tersoff, J. & Hamann, D. R. Theory and application for the scanning tunneling microscope. *Phys. Rev. Lett.* **50**, 1998–2001 (1983).
